# Chronological change of gallbladder fossa nodularity in the liver as observed in patients with alcoholic liver disease: cross-sectional and longitudinal observation

**DOI:** 10.1007/s11604-025-01741-5

**Published:** 2025-02-12

**Authors:** Keisuke Sato, Eiko Hisatomi, Shinji Tanaka, Nahoko Goto, Ryo Murayama, Yukihisa Takayama, Kengo Yoshimitsu

**Affiliations:** Department of Radiology, Faculty of Medicine, Fukuoka University, 7-45-1 Nanakuma Jonan-ku, Fukuoka City, 814-0180 Japan

**Keywords:** Alcoholic liver disease, Gallbladder fossa nodularity, Chronological change, Expanded gallbladder fossa, Gadoxetate-enhanced MRI, MR elastography, Extracellular volume fraction

## Abstract

**Purpose:**

To confirm the concept that gallbladder fossa nodularity (GBFN) as observe in patients with alcoholic liver disease (ALD) develops in a biphasic fashion as the cirrhotic process progresses, by clarifying the sequential or chronological change of GBFN both in cross-sectional and longitudinal analyses.

**Materials and methods:**

We retrospectively recruited 52 ALD patients who had both quadruple phase CT and gadoxetate-enhanced MRI (EOB-MRI) within 6 months between 2013 and 2020, and GBFN were morphologically classified into grades 0–3, as previously described. As a cross-sectional study, correlation coefficients (rho values) between mALBI grades and GBFN grades were compared for monophasic vs biphasic models. Biphasic models were defined based on the median values of liver stiffness as measured by MR elastography and extracellular volume fraction as calculated from CT data. Similar analysis was done for GBFN signal intensity on hepatobiliary phase of EOB-MRI (HBP-SI). As a longitudinal study, we recruited patients for whom at least 3-year follow-up of GBFN were available using any CT or MR imaging examination.

**Results:**

As for cross-sectional study, the rho values for the biphasic model were significantly larger than those for the monophasic model, both for GBFN grades and HBP-SI (*p* < 0.01). As for the longitudinal study, 10 patients were available, 6 of whom showed downgrading of GBFN as the cirrhotic change progressed.

**Conclusion:**

Our cross sectional and longitudinal analyses suggested GBFN would develop in a biphasic pattern both on morphology and HBP-SI as the cirrhotic process progresses.

**Supplementary Information:**

The online version contains supplementary material available at 10.1007/s11604-025-01741-5.

## Introduction

Recently, it has been reported that nodularity of the gallbladder fossa (GBFN) as observed typically in patients with alcoholic liver disease (ALD) may represent spared area from portal venous perfusion containing alcohol due to cholecystic venous drainage (CVD) [1–4], and escaping from alcohol-induced fibrotic and atrophic change of the surrounding background liver (BGL) parenchyma [5]. Pathologically, it has been shown that GBFN consisted of unusually large-sized pseudo-lobules in contrast to small-sized pseudo-lobules that are known to be characteristic of alcoholic liver cirrhosis, which may as well be called as “pseudo-hyperplasia” [5–7]. Also shown was the positive correlation between GBFN grades and mALBI grades [8, 9], suggesting GBFN becomes more and more prominent as cirrhotic change progresses [5, 6]. This concept was further confirmed by showing the areas of GBFN tended to show relatively high signal on the hepatobiliary phase images (HBP) of gadoxetate-enhanced MRI, and relatively low extracellular volume fraction (ECV) as calculated from routine CT data, as compared to background liver (BGL) [6].

Thus, evidences have been accumulated to support the concept that GBFN represents spared, and relatively normal, “pseudo-hyperplastic” liver parenchyma due to CVD in contrast to the fibrotic and atrophic BGL caused by exposure to the alcohol-containing portal perfusion.

However, simply thinking, if GBFN develops according to the progression of cirrhosis, which would be contradictory to the famous “expanded gallbladder fossa” sign [10–12]. Indeed, we have noticed that grade 3 GBFN, which should be the extreme form of GBFN, are not necessarily observed in the extreme phase or end-stage cirrhosis, namely, in mALBI grade 3 patients: actually, in the previous study [6], four grade 3 GBFN were observed in 1/2/1/0 patients with mALBI grades 1/2a/2b/3, respectively. Similarly, grade 3 GBFN not necessarily show high signal intensity on HBP relative to BGL (HBP-SI): 4 grade 3 GBFN in the previous study [6] exhibited HBP-SI of iso-/slightly high/high intensity in 1/2/1 patients, respectively. Based on these observations, we hypothesized that grade 3 morphology or high signal of GBFN does not represent final or end-stage cirrhosis, but represents a mid-stage or transitional stage of the cirrhotic process: namely, GBFN would show a biphasic development pattern during the progression of cirrhotic change. To confirm this hypothesis, we attempted to clarify the sequential or chronological change of GBFN according to the progression of cirrhosis, from both cross-sectional and longitudinal approaches in this study.

## Materials and methods

### Patients

**Fig. 1:**
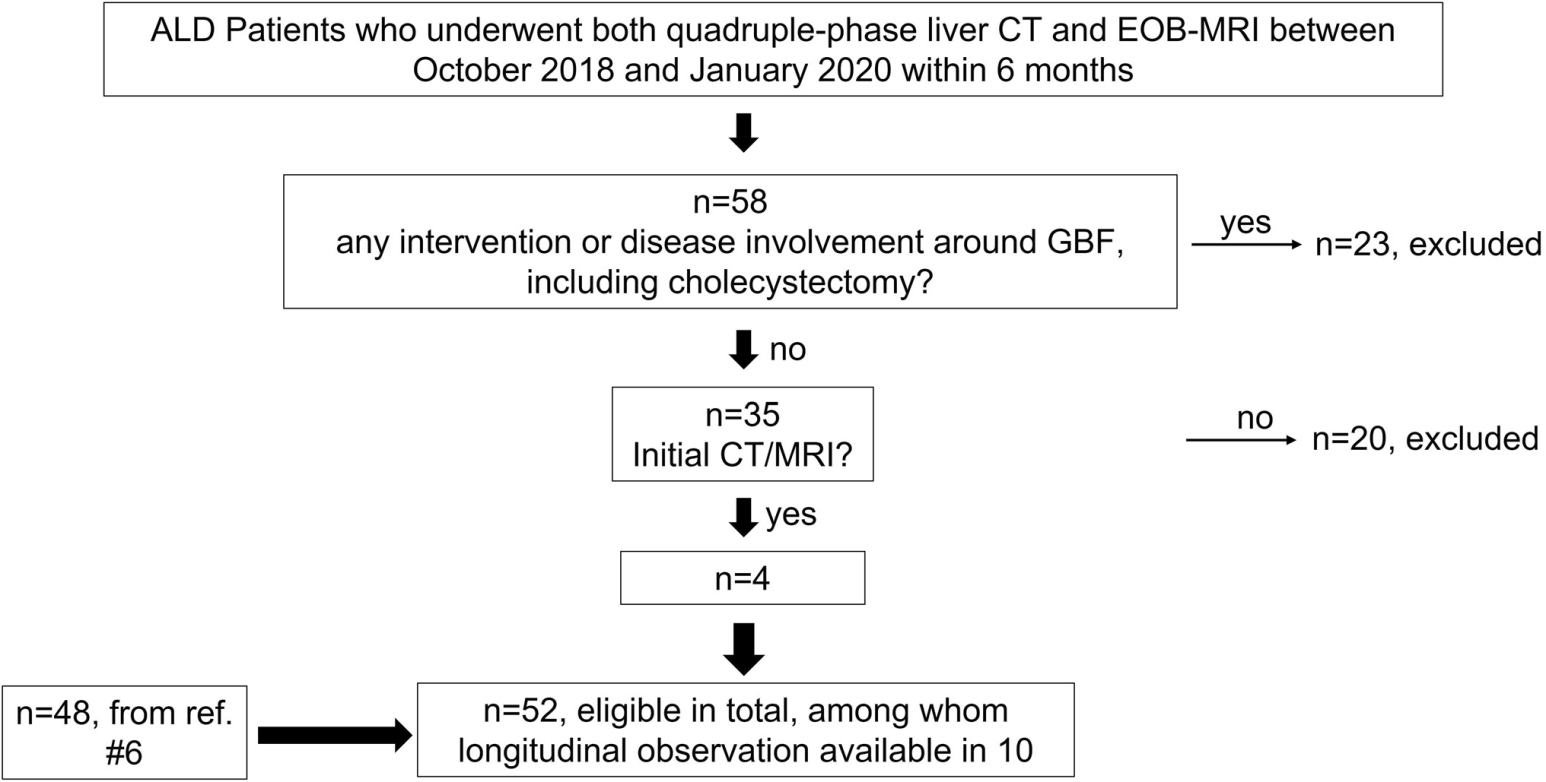
Patient inclusion flow chart. *ALD* alcoholic liver disease, *EOB-MRI* gadoxetate-enhanced MRI, *GBF* gallbladder fossa

In addition to the 48 patients in the previous study [6], the study period for which was between 2013 and 2018, we extended the survey period to January 2020, and ALD patients for whom both quadruple CT and EOB-enhanced MRI were obtained within six months were further recruited. Patient selection criteria are similar to the previous studies [5, 6]: roughly speaking, clinical diagnosis of ALD was made for patients with liver dysfunction without serological evidence of hepatitis B or C infection, or anti-mitochondrial or anti-nuclear antibodies, and with a history of habitual alcohol overconsumption, based on the criteria defined by Japanese Society of Biomedical Research of Alcohol [13], that is, briefly, 60 g/day and 40 g/day or more alcohol consumption for men and women, respectively. Among those, patients who had had cholecystectomy and hepatectomy, apparent acute cholecystitis or cholangitis, hepatocellular carcinoma (HCC) involving gallbladder fossa, and tumor thrombus involving the 1st order branch or main trunk of the portal vein were excluded. Institutional review board approved this study and waived obtaining informed consent from the patients because of its retrospective nature, and this study was performed per the ethical standards as laid down in the 1964 Declaration of Helsinki. Patient selection flow chart is shown in Fig. 1.

### CT protocol

For cross-sectional study, two CT equipment were used, with 2 mm thickness reconstruction and four phase dynamic scanning. The details of CT protocol are described in the previous studies [5, 6], and provided in the supplementary materials. Portal venous phase was additionally reconstructed along the coronal plane with 2 mm contiguous slice thickness. After obtaining unenhanced images, 600 mgI/kg iodine contrast medium (Iopamiron 370, Bayer Health Care, Osaka, Japan) was injected for 30 s at a variable injection rate, and arterial dominant phase images were obtained using bolus tracking method, followed by portal dominant phase at 60 s, and equilibrium phase images at 240 s after the commencement of contrast medium injection. ECV map was generated using these CT data, according to the previously reported methods [6, 14–16].

For the longitudinal study, CT images obtained with protocols other than quadruple phase protocol as mentioned above, including non-contrast CT with 5 mm thickness reconstruction, were also included for review, to simply assess the morphological change of GBFN.

### MR protocol

The details of the MR protocol are described in the previous study [6], and also provided in the supplementary materials (Table S1). The MR equipment used was a 3 T clinical unit (Discovery 750W, GE, Milwaukee, USA) with a 32-element phased-array coil. MR elastography (MRE) was routinely included in liver MR protocol in our institution, and obtained according to the previously described methods [17–20] before contrast enhancement. Roughly, a 2D spin-echo echo-planar MRE sequence was acquired using a 42 cm field-of-view and magnitude and phase difference wave images were generated. Four slices were obtained including the level of the hepatic hilum under 16-s breath-holding. Liver stiffness in kPa was measured by placing as large a region-of-interest as possible in the right lobe [17–20], and averaged value of the four slices was considered to represent the liver stiffness of the individual. Liver stiffness values were adopted from the radiological reports recorded at the time of interpretation and were not specifically measured again for the purpose of this study.

### Assessment

#### Cross-sectional analyses

GBFN grades were defined according to the previous studies [5, 6] as shown in the supplementary materials (Fig. S1). Based on our observation as mentioned earlier, we hypothesized that GBFN morphological change would occur sequentially in a biphasic fashion as the cirrhotic change progresses as follows; grade 0, grade 1, grade 2, grade 3, grade 2 (name this as grade 2’ to discriminate from the earlier one), grade 1 (name this as grade 1’), and grade 0 (similarly grade 0’). To distinguish grades 0’−2’ from grades 0–2, we used the median value of liver stiffness as measured by MRE and that of ECV of BGL (ECV_BGL_) as well; in other words, GBFN grades 0–2 with liver stiffness larger than its median value were defined as grades 0’, 1’, and 2’: similarly, GBFN grades 0–2 with ECV_BGL_ larger than its median value were defined as grades 0’, 1’, and 2’. We compared the correlation coefficients, namely rho values, of Spearman’s rank correlation test, between mALBI grade vs conventional GBFN grade sequence (monophasic model, from grade 0 to 3), and between mALBI grade vs new GBFN grade sequence (biphasic model, from grade 0 to 3, and further to 2’, 1’ and 0’). Similarly, we hypothesized that HBP-SI would sequentially change in a biphasic fashion from iso-, slightly high, high intensity, and then, slightly high (named this as slightly high’), and to iso- (name this as iso’-) intensity. Slightly high’ and iso’-intensity were defined as slightly high or iso-signal intensity which has higher kPa or ECV_BGL_ than their median values. We compared the rho values between mALBI grade vs conventional HBP-SI sequence (monophasic model; iso-, slightly high, and high signal intensity), and between mALBI grade vs new HBP-SI sequence (biphasic model; iso-, slightly high, high, slightly high’, and iso ‘-signal intensity).

All qualitative and quantitative assessment (ECV measurement) results obtained in the previous study [6] were used as they were, and re-assessment was not performed for this cohort (48 patients). Regarding inter-observer concordance assessment, one of the authors (ST) performed ECV measurement for the remaining 28 patients, because inter-observer agreement was assessed only for the first 20 patients in the previous study [6]. For the additionally found patients, two of the authors (KS and ST, same radiologists who interpreted in the previous study [6]) reviewed the images independently first, and the disagreement was solved by consensus: both of them measured ECV on ECV map independently, according to the method as described in the previous study [6]. More specifically, as for HBP-SI assessment, both axial and coronal images were reviewed, and a higher signal on either plane was adopted to represent the patient; as for ECV_BGL_ measurement, the anterior, posterior, and medial segment were used. The average of the measured values by the two radiologists was used for further analyses. Agreement assessment between the two radiologists was finally performed for the total patients, including the previous 48 patients [6] and additionally found patients.

#### Longitudinal analysis

 For the patients for whom more than three-year follow-up were available, changes in the GBFN grades were recorded, in correlation with the change in mALBI grades/scores at the time of examinations. For this longitudinal study, CT or MR images were searched for from even before, or after the study period (2013 through 2020), and non-contrast CT or MR images were included for the purpose of morphological assessment. Among the available follow-up images, study coordinator (KY) selected relevant CT or MR studies, which were assessed by two radiologists (KS, ST) independently first, and disagreement was resolved by consensus. Final morphological interval change of GBFN per patient was determined either by CT or MR images.

### Statistics

Inter-observer concordance for qualitative and quantitative assessments was assessed with kappa statistics, and intra-class coefficient (ICC), respectively. Correlation between non-parametric variables was assessed using Spearman’s rank correlation tests. For comparison of rho values or correlation coefficients, Fisher’s Z-transformation was used. P values less than 5% were considered as statistically significant. All statistical analyses were performed using JMP Pro14.3.0 (SAS corporation, Cary, USA).

## Results

### Patients

In addition to the 48 patients who were included in the previous study [6], we found 4 more patients from the extended recruiting period. Thus, a total of 52 patients who have both quadruple CT and EOB-MRI within six months were analyzed in this study, the demographic data of whom are shown in Table 1. The kappa value for the two radiologists in the assessment of GBFN grades was 0.70, suggesting good agreement. That for signal intensity of GBFN on HBP was 0.85, showing excellent agreement.

**Table 1:** Patient demographic data and other information

Sex (M:F)	46:6
Age (Av ± SD, range)	60.6 ± 11.0 (28—83)
mALBI grade (1/2a/2b/3)	16/10/23/3
CP grade*score (A5/A6/B7/B8/B9/C10)	22/16/5/4/4/1
GBFN grade (0/1/2/3)	11/17/19/5
Interval between CT and MRE (mo)	3.2 ± 0.6 (1—6)
HBP signal intensity (iso/ slightly high/ high)	31/ 18/ 3
Liver stiffness (kPa, Av ± SD, range)	6.9 ± 3.2 (1.7–12.8, median 6.5)
ECV of GBFN (%, Av ± SD, range)	31.2 ± 7.9 (14.6–50.4, median 30.7)
ECV of BGL (%, Av ± SD, range)	36.2 ± 8.0 (22.6–53.2, median 35.8)
ECV ratio (Av ± SD, range)	0.88 ± 0.19 (0.53–1.36, median 0.88)

*M/F* male/female, *Av/SD* average/ standard deviation, *mALBI* modified albumin-bilirubin, *CP* Child -Pugh, *GBFN* gallbladder fossa nodularity, *MRE* MR elastography, *HBP* hepatobiliary phase, *ECV* extracellular volume fraction, *BGL* background liver. ECV ratio is defined as ECV of GBFN divided by that of GBL

### Cross-sectional analysis

The median value of the stiffness of the liver was 6.5 kPa (Table 1), which was used to define the biphasic model of GBFN grades and HBP-SI sequential change. There were 10/11/3/5/16/6/1 patients for GBFN grade 0/1/2/3/2’/1’/0’, and 19/7/3/9/14 patients for HBP-SI iso-/slightly high/high/slightly high’/iso’-intensity, respectively. The median value of ECV_BGL_ turned out to be 35.8% (Table 1), by which biphasic GBFN grades and HBP-SI sequential change were defined. There were 12/6/8/5/12/8/1 patients for GBFN grade 0/1/2/3/2’/1’/0’, and 20/8/3/7/14 patients for HBP-SI iso-/slightly high/high/slightly high’/iso’-intensity, respectively.

**Fig. 2:**
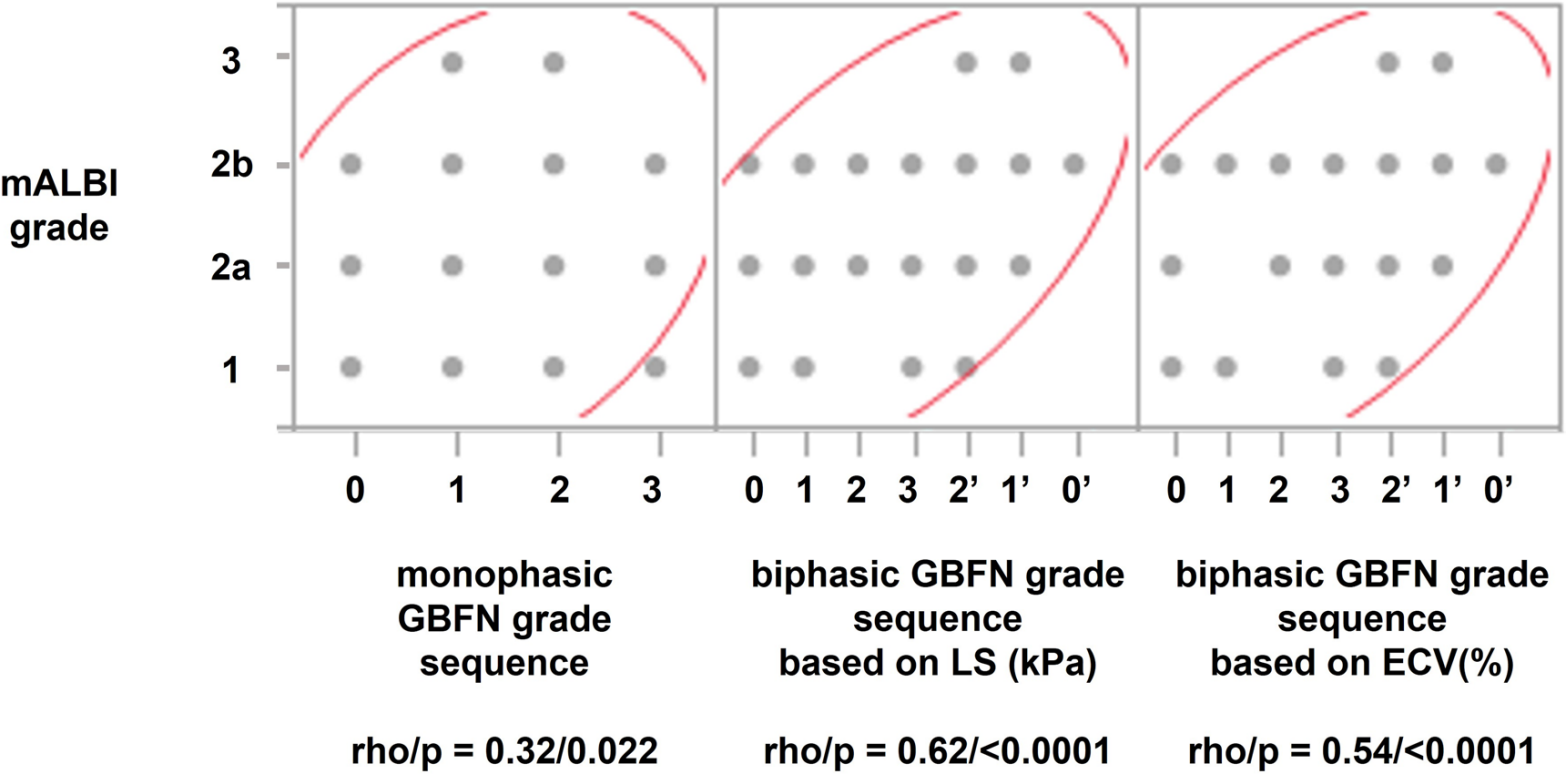
Correlation between modified ALBI (mALBI) grades and gallbladder fossa nodularity (GBFN) grades in three models. Although the correlations were positively significant in all three models, the rho values at Spearman’s rank correlation test for biphasic models were significantly larger than that of the conventional monophasic model (0.32) both for that sorted by liver stiffness (LS) in kPa (0.62, *p* < 0.01) and that sorted by extracellular volume fraction (ECV) in % (0.54, *p* < 0.05)

**Fig. 3:**
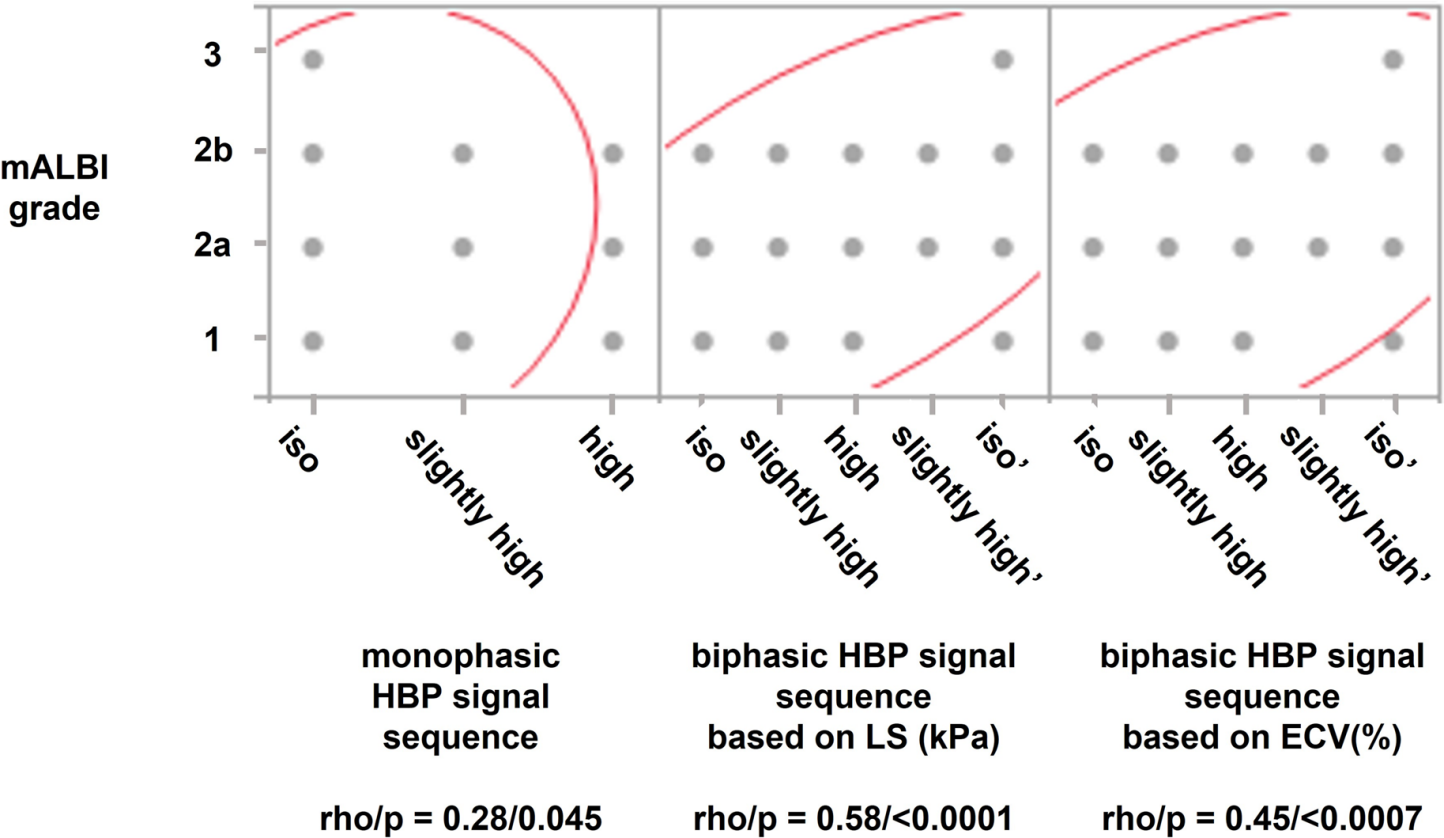
Correlation between modified ALBI (mALBI) grades and relative signal intensity of gallbladder fossa nodularity (GBFN) at the hepatobiliary phase of gadoxetate-enhanced MRI (HBP-SI) in three models. Although the correlations were positively significant in all three models, the rho values at Spearman’s rank correlation for biphasic models were larger than that of conventional sequence (0.28) for that sorted by liver stiffness (LS) in kPa (0.58, *p* < 0.01) with statistical significance, and for that sorted by extracellular volume fraction (ECV) in % (0.48, *p* = 0.17) without statistical significance

ICC of ECV measurement by the two radiologists was 0.78, suggesting good agreement. Correlation coefficients, namely rho values, between GBFN grades vs mALBI grades, or those between HBP-SI and mALBI grades, were all positively significant, either for monophasic or biphasic model (*p* < 0.05, Spearman’s rank correlation). However, those for the biphasic model were significantly larger, for both models sorted by kPa (0.62, *p* < 0.01, Fisher’s Z-transformation test) and ECV_BGL_ (0.54, *p* < 0.05, Fisher’s Z-transformation test), than that of monophasic GBFN grade models (0.32) (Fig. 2). Similarly, rho value for biphasic HBP-SI model was significantly larger for that sorted by kPa (0.58, *p* < 0.05, Fisher’s Z-transformation test) than that of monophasic GBFN grade sequence (0.28), but the difference did not reach statistical significance for that sorted by ECV_BGL_ (0.45, *p* = 0.17, Fisher’s Z-transformation test) (Fig. 3). These results would strongly support the appropriateness of the biphasic models, as the order of sequential change in morphology and HBP-SI of GBFN. Actual number of observations for both models are shown in the supplementary materials (Fig. S2).

### Longitudinal analysis

There were 10 patients, including 8 men and 2 women, for whom more than three-year follow-up were available: ages at the time of an initial set of four-phase CT and EOB-MRI ranged from 28 to 69 years old, with a median of 54.5: follow up period ranged from 3 to 12 years, with a median of 5.5. For these 10 patients, 34 CT examinations (from 2 to 5, with a median of 3, per patient) and 7 MRI (2, 2, and 3, per patient) examinations were selected for review in total, for which kappa value between the two radiologists’ interpretation for GBFN grades was 0.75, suggesting good agreement. Of these 10 patients, liver function (mALBI grade) deteriorated in 4, and was stable in 6, during the follow-up periods, and none showed mALBI grade improvement. In 6 patients out of 10, GBFN morphologically down-graded (from grades 2 or 3 to grades 1 or 0), and in only one up-graded (from grade 1 to 3). Details are shown in Table 2.

**Table 2:** Details of the longitudinal change of the 10 patients for whom more than 3-year follow up was available

Liver function GBFN grade	deteriorate	No change	improve
Up-grade	0	1 G1 → G3: 2b/ 5yrs¶	0
No change	2 G1: 2b → 3/ 3yrs G3: 2a → 2b/ 6yrs	1 *G2: 2b/ 3yrs	0
Down-grade	2 G3 → G1: 2a → 3/ 8yrs† G2 → G1: 2a → 3/ 3yrs	4 G2 → G1: 2b/ 7yrs G2 → G1: 2b/ 12yrs *G2 → G0: 3/ 7trs G3 → G1: 2b/ 8yrs‡	0

*GBFN* gallbladder fossa nodularity, *G* grade, *yrs* years, *: female patient

**Fig. 4:**
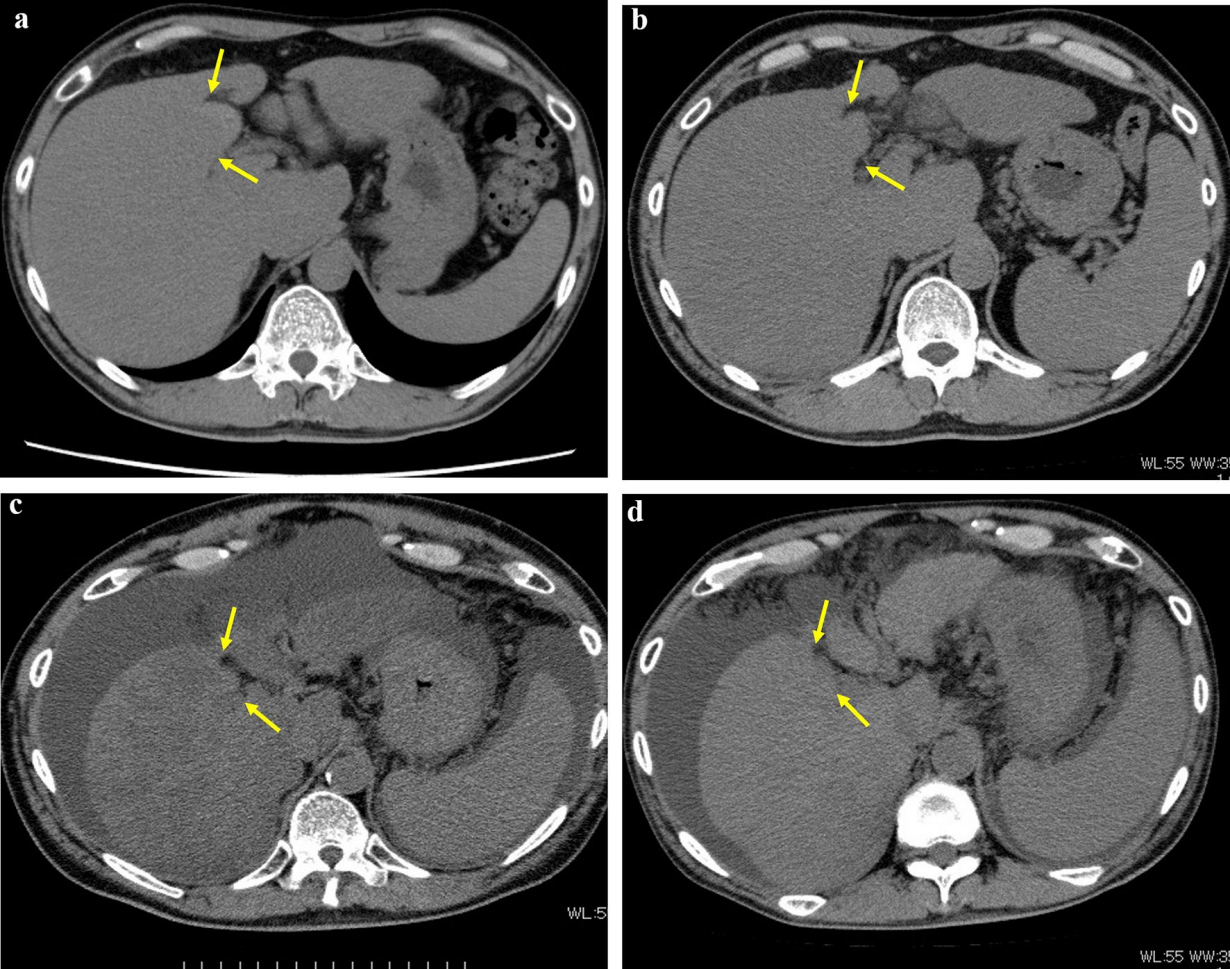
Longitudinal change of gallbladder fossa nodularity (GBFN) in a 47-year-old man with alcoholic liver disease. **a** Non-contrast CT reveals grade 3 GBFN. ALBI score and modified ALBI grade were − 2.4 and grade 2a. **b** Non-contrast CT obtained 3 years later reveals less prominent GBFN, but still grade 3 was assigned. ALBI score and modified ALBI grade were − 1.8 and grade 2b. **c** Non-contrast CT obtained 4 years later (7 years from 4a) reveals markedly shrunk GBFN, for which grade 2 was assigned. Moderate amount of ascites and fatty liver is apparent. ALBI score and modified ALBI grade were − 0.8 and grade 3. **d** Non-contrast CT obtained one year later (8 years from 4a) reveals further shrinkage of GBFN, for which grade 1 was assigned. Moderate amount of ascites is still present and fatty liver improved. ALBI score and modified ALBI grade were − 1.3 and grade 3, respectively, suggesting substantial deterioration of liver function since 4a. Note prominent splenomegaly and less prominent caudate lobe enlargement as compared to 4a

^†^: patient in Fig. 4

**Fig. 5:**
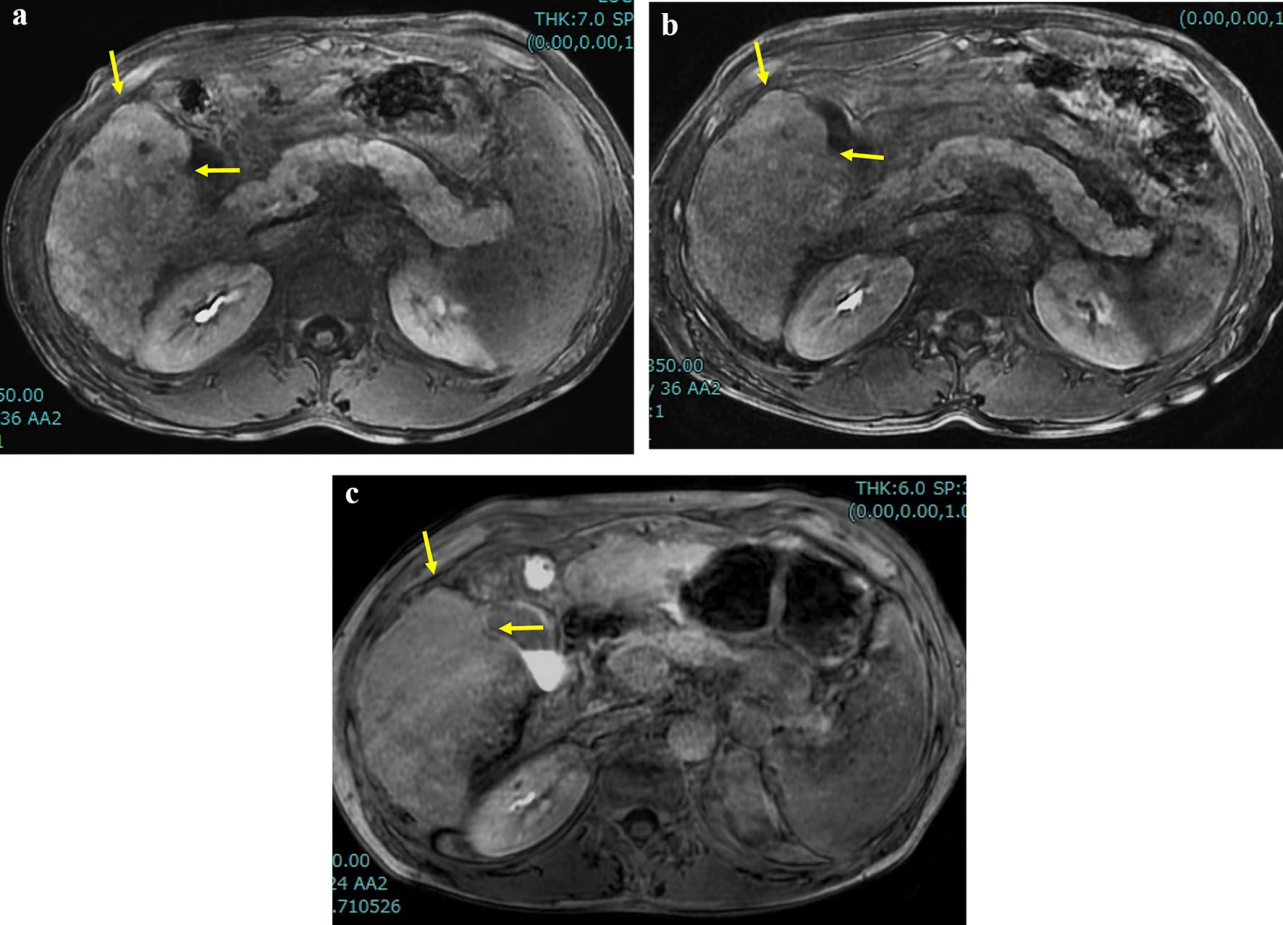
Longitudinal change of gallbladder fossa nodularity (GBFN) in 42-year-old man with alcoholic liver disease. **a** Hepatobiliary phase (HBP) of gadoxetate-enhanced MRI revealed grade 2 GBFN (arrows). Note slightly high signal intensity at the peritoneal side of GBFN. ALBI score and modified ALBI grade were − 1.7 and grade 2b. **b** HBP image one year later reveals less prominent GBFN, but grade 2 (arrows) was again assigned. Note the relatively high signal on the peritoneal side as observed in 5a is less prominent. ALBI score and modified ALBI grade were − 1.8 and grade 2b. **c** HBP image five years later (6 years from 5a) reveals grade 1 GBFN (arrows), exhibiting mostly iso-signal intensity as compared to the surrounding liver parenchyma. ALBI score and modified ALBI grade were − 1.4 and grade 2b, respectively, suggesting slight progression of liver dysfunction since 5a

^‡^: patient in Fig. 5: This patient had a CT before 7a and another CT after 7c, therefore, total follow-period was 8 years

**Fig. 6:**
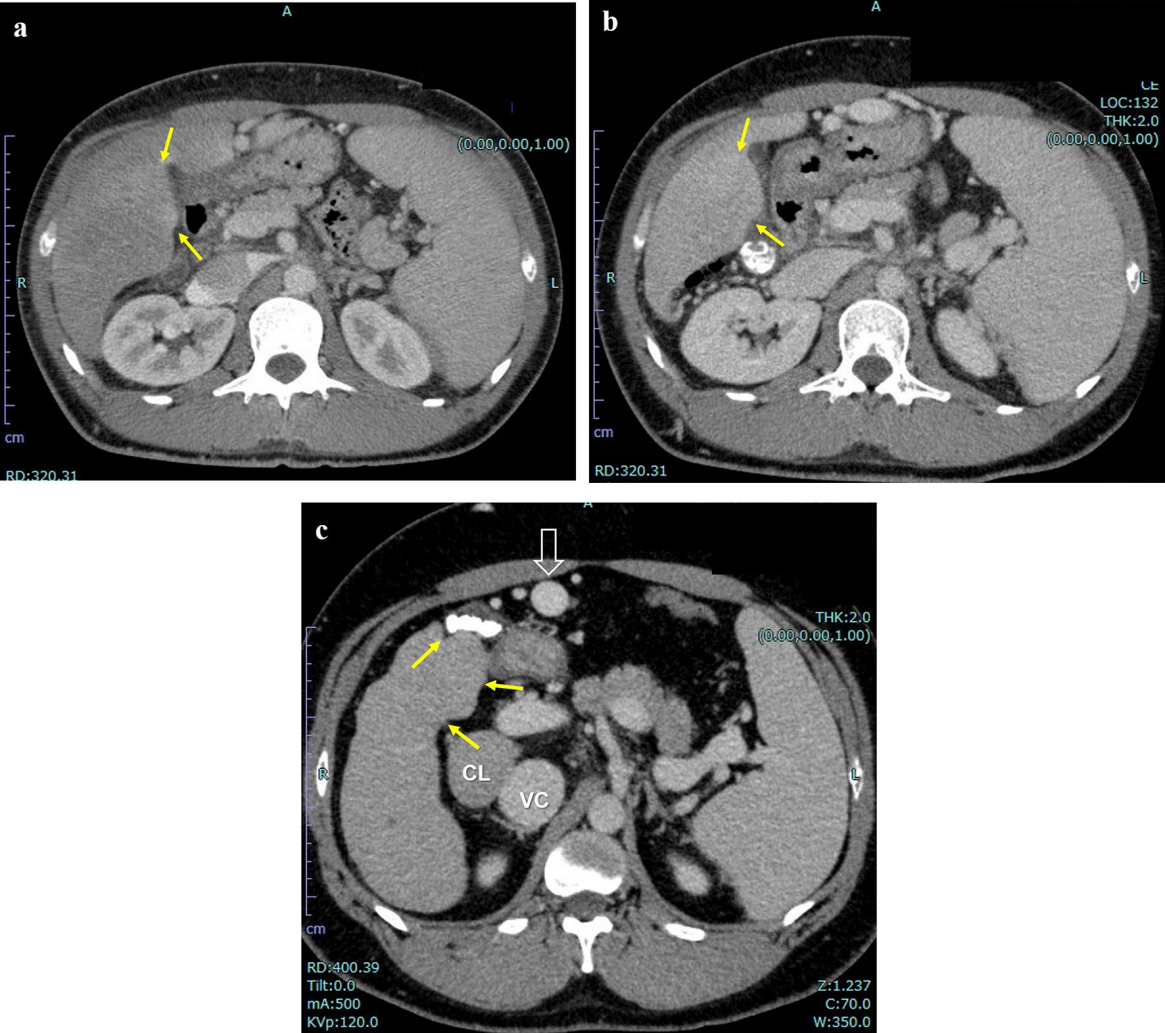
Longitudinal change of gallbladder fossa nodularity (GBFN) in 28-year-old man with alcoholic liver disease. **a** Portal phase of contrast-enhanced CT reveals hepatosplenomegaly and grade 1 GBFN (arrows). Clinically, this patient was in the acute phase of alcoholic liver injury at this time. ALBI score and modified ALBI grade were − 2.1 and grade 2b. **b** Portal phase of contrast-enhanced CT one year later again revealed grade 1 GBFN (arrows). ALBI score and modified ALBI grade were − 1.5 and grade 2b. **c** Portal phase of contrast enhanced CT 4 years later (5 years from 6a) revealed grade 3 GBFN (arrows). ALBI score and modified ALBI grade were − 1.4 and grade 2b, respectively, suggesting deterioration of liver function since 6a. Note enlarged caudate lobe (CL), irregular surface of the lateral aspect of the right lobe, enlarged paraumbilical vein (empty arrow), and enlarged inferior vena cava (vc) due to massive splenorenal shunt (not shown). Also note this slice is several cm cephalad to 6a or 6b, indicating marked atrophic change of background liver and progression of cirrhotic change

^¶^: patient in Fig. 6

Information within the parentheses indicates those for each patient. The left-side, the center, and the right-side information within parentheses represent GBFN grade, liver function as expressed by modified ALBI grade, and duration of follow-up in years, respectively. For example, “G3 → G1: 2a → 3/ 8yrs” indicates GBFN grade changed from G3 to G1, and mALBI grade changed from 2a to 3, over 8-year follow-up

Representative cases are shown in Figs. 4, 5, 6.

## Discussion

In this study, we hypothesized that GBFN would show a sequential morphological change in a biphasic fashion, namely, from grade 0 to 3 in ascending order first, and then, from grade 3 to 0 in descending order: sequential change of HBP-SI of GBFN would also be biphasic in pattern, from iso-intensity, to slightly high, high, and then, slightly high, and finally to iso-intensity, as the cirrhotic change of the liver progresses.

In the cross-sectional study, we found that rho values at Spearman’s rank correlation test between mALBI grades vs biphasic sequential order of GBFN grades were significantly larger than that of monophasic order, in both models sorted by kPa and ECV_BGL_; similarly, rho values for biphasic sequential order of HBP-SI were larger than that for monophasic order, with statistical significance in the model sorted by kPa, but without, in the model sorted by ECV_BGL_. These may support our hypothesis that GBFN develops not simply in a monophasic fashion, but in a rather complicated biphasic fashion both in morphological grade and HBP-SI. In the longitudinal study, the above hypothesis was partially confirmed, in that morphological down-grading was observed as the cirrhotic change progresses to the end stage (Fig. 4); only one showed morphological up-grading as liver function deteriorate (Fig. 6), and unfortunately, there was no single individual revealing biphasic sequential change in GBFN morphology in this study.

**Fig. 7:**
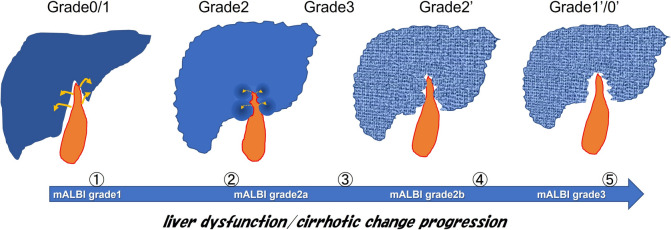
Schematic presentation of hypothetic sequential change of gallbladder fossa nodularity (GBFN). ➀ Cholecystic venous drainage (CVD, curved arrows) perfuses liver parenchyma around GBF, which is spared from portal venous perfusion containing alcohol. ➁ Alcohol causes fibrotic/atrophic/cirrhotic change in background liver (BGL), leaving GBF areas as relatively normal or pseudohyperplastic parenchyma, exhibiting GBFN. While cirrhotic change progresses, GBFN becomes more and more prominent. On the other hand, tissue pressure in BGL would elevate at the same time, making it difficult for CVD to perfuse liver tissue around GBF. This would lead to diminishment in CVD inflow (dotted curved arrows). ➂ Diminished CVD inflow to the liver would result in less prominent GBFN. ➃ Then, portal venous blood may start to perfuse the GBF area, which would be exposed to the effect of alcohol, resulting in fibrotic/atrophic/cirrhotic change, in a similar fashion as that of BGL. ➄ “Expanded GBF sign”may be observed at this stage. *mALBI* modified ALBI

This whole process of chronological or sequential GBFN change can be explained as follows (Fig. 7): at the early stage of ALD, CVD perfuses the GBF area which would be spared from fibrotic and atrophic influence caused by alcohol, resulting in relatively normal or preserved liver tissue at GBF in contrast to the damaged BGL, which may be called “pseudo-hyperplasia”. While the cirrhotic change progresses, the tissue pressure in BGL may elevate, which would hamper CVD to perfuse the areas of GBF. As CVD inflow to GBF areas thus diminishes, alcohol-containing portal venous blood may start to perfuse these areas, which would result in fibrotic and atrophic change similar to BGL. Then, GBFN may disappear at this stage, where so-called “expanded GBF” sign [10–12] may be established.

There are several limitations of this study. Due to its retrospective nature, there could be an inherent bias in the patient selection. That there was not enough number of patients for whom longitudinal observation was available, particularly with EOB-MRI, may be another limitation. Lack of pathological proof might be an additional limitation, however, considering the patients status in this cohort, obtaining pathological specimens from these patients may not be ethically justified. Another limitation may include the subjective way of assessing the grades of GBFN, HBP signal intensity, or ECV measurement. However, the kappa values or intra-class correlation coefficient in the previous [6] (48 patients) and the current study (52 patients for cross sectional, and 10 patients for longitudinal study, respectively) suggested agreement between the two readers was reasonably high.

In conclusion, GBFN in ALD patients may show the chronological or sequential change in a biphasic pattern both in morphology and in HBP-SI, as the cirrhotic change progressed in the liver. This knowledge may be useful for radiologists when interpreting radiological images of CLD patients in daily clinical practice.

## Supplementary Information

Below is the link to the electronic supplementary material.

Supplementary file1 (DOCX 454 KB)

Supplementary file2 (DOCX 33 KB)
