# Supplementary material for: Chronological change of gallbladder fossa nodularity in the liver as observed in patients with alcoholic liver disease: cross-sectional and longitudinal observation
Source: Jpn J Radiol. 2025 Feb 12;43(6):967–76. doi: 10.1007/s11604-025-01741-5 (PMC12125056; doi:10.1007/s11604-025-01741-5)
Supplement: Supplementary file 1 — Supplementary file1 (DOCX 454 KB) [file 11604_2025_1741_MOESM1_ESM.docx]

Supplementary materials

**CT equipment, and parameters**

1. An area-detector CT (Aquilion ONE ViSION Edition, Canon Medical Systems, Tokyo, Japan), and scanning parameters were as follows: 0.5 mm x 80 row, 120 kVp, three-dimensional auto-exposure control (Volume EC: SD12@5mm), 0.5 sec/rotation, 0.813 beam pitch, 512 x 512 matrix, 300-350 mm field-of-view, and 2mm reconstruction. Noise reduction was achieved by a hybrid iterative reconstruction (ADIR 3D Weak).
2. A 64-row multi-detector CT (Aquilion 64, Canon Medical Systems, Tokyo, Japan), with parameters shown below: 0.5 mm x 64 row, 120 kVp, three-dimensional auto-exposure control (Volume EC: SD12@5mm), 0.5 sec/rotation, 0.828 beam pitch, 512 x 512 matrix, 300-350 mm field-of-view, and 2mm reconstruction (filtered back projection). Portal venous phase was additionally reconstructed along the coronal plane with 2mm contiguous slice thickness in either CT.

**Table S1 MR protocols and parameters**


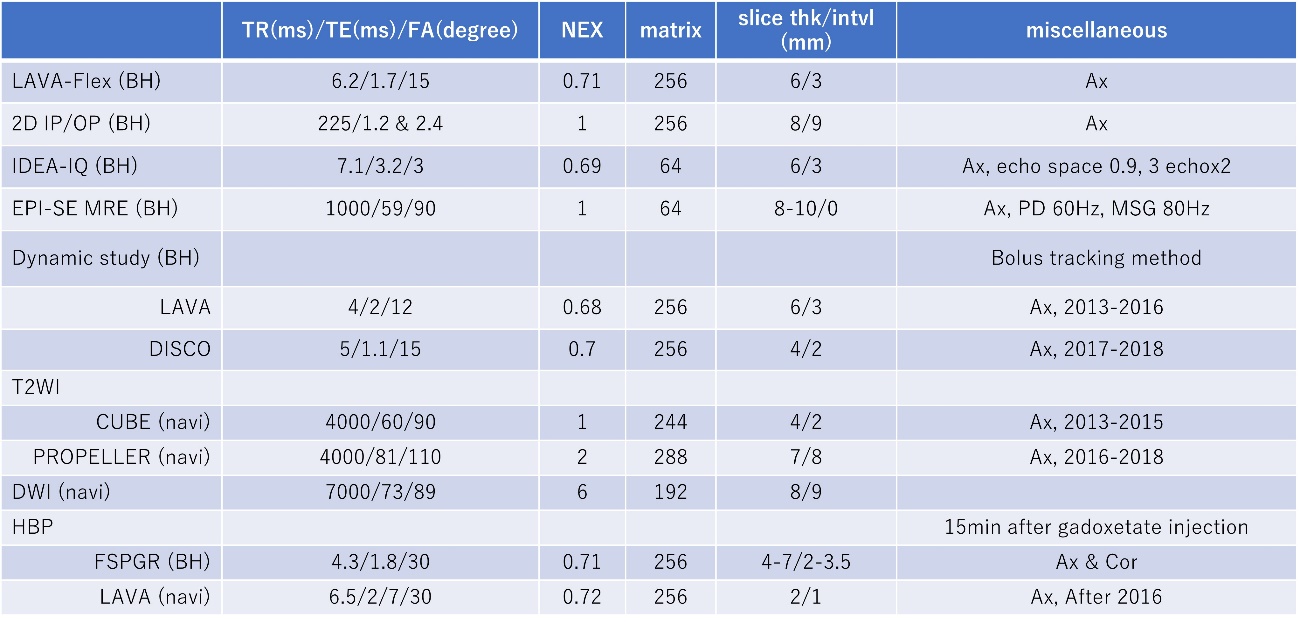


TR: repetition time, TE: echo time, NEX: number of excitation, thk: thickness, intvk: interval, LAVA: liver acquisition with volume acceleration, LAVA-Flex: 3D T1-weighted image using two-point Dixon method, BH: breath hold, Ax: axial, IP: in phase, OP: out of phase, IDEAL: Iterative decomposition of water/fat using echo asymmetry and least squares estimation, IDEAL-IQ: 3DT1-weighted image using 6-point Dixon method, EPI: echo planar image, SE: spin-echo, PD: passive driver, MSG: motion sensitizing gradient, DISCO: differential subsampling with Cartesian ordering, T2WI: T2-weighted image, CUBE: 3D fast spin-echo with variable flip angle, navi: navigation, PROPELLER: periodically rotated overlapping parallel lines with enhanced reconstruction, DWI: diffusion-weighted image, HBP: hepatobiliary phase, FSPGR: fast spoiled gradient-echo sequence, Cor: coronal

**Fig. S1** Scheme of the grades of gallbladder fossa (GBF) nodularity. Grade 0 represents flat surface the gallbladder (GB) fossa without notch or protrusion, which is not shown here.

Fig. 2A Grade 1. Minimal protrusion with slight notch at GBF.

Fig. 2B Grade 2. Slight protrusion with notches, the degree of which is between Grade 1 (2A) and Grade 3 (2C).

Fig. 2C Grade 3. Apparent nodularity of GBF.


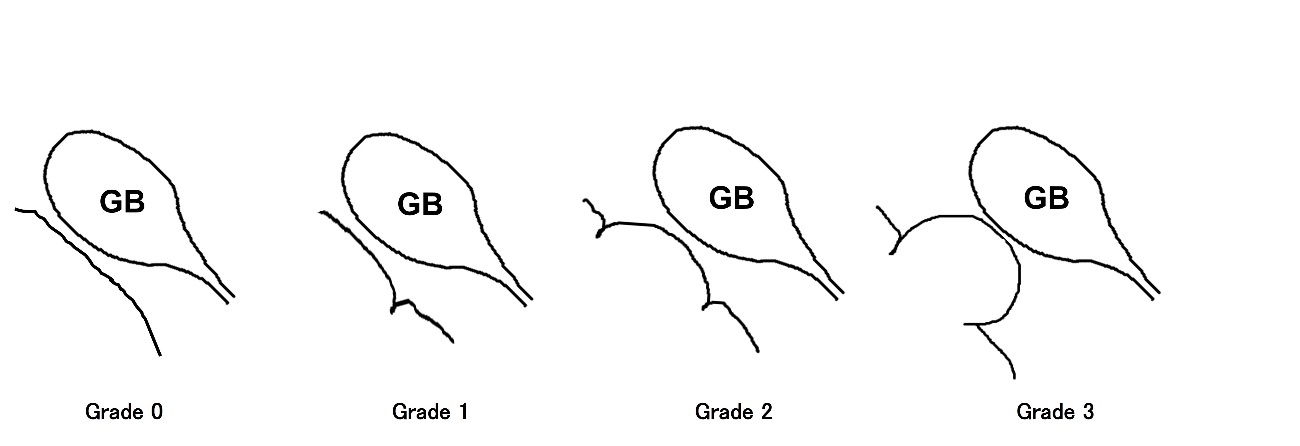


**Fig. S2** Contingency tables for Fig.s 2 and 3, showing actual number of patients

S2-A mALBI grade vs gallbladder fossa nodularity (GBFN) grades


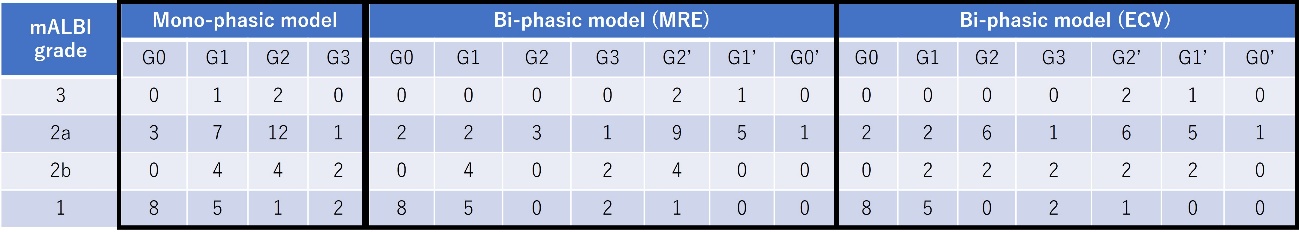


G indicates GBFN grade.

S2-B mALBI grade vs hepatobiliary phase signal intensity (HBP-SI)


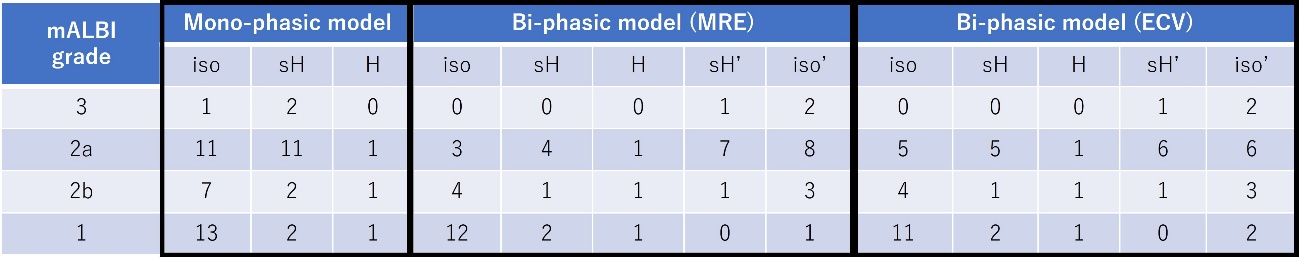


iso: iso-intensity, sH: slightly high, H: high
