# Supplementary material for: Chronological change of gallbladder fossa nodularity in the liver as observed in patients with alcoholic liver disease: cross-sectional and longitudinal observation
Source: Jpn J Radiol. 2025 Feb 12;43(6):967–76. doi: 10.1007/s11604-025-01741-5 (PMC12125056; doi:10.1007/s11604-025-01741-5)
Supplement: Supplementary file 2 — Supplementary file2 (DOCX 33 KB) [file 11604_2025_1741_MOESM2_ESM.docx]

Table 1 Patient demographic data and other information

| Sex (M:F) | 46:6 |
| --- | --- |
| Age (Av ± SD, range) | 60.6 ± 11.0 (28 - 83) |
| mALBI grade (1/2a/2b/3) | 16/10/23/3 |
| CP grade*score (A5/A6/B7/B8/B9/C10) | 22/16/5/4/4/1 |
| GBFN grade (0/1/2/3) | 11/17/19/5 |
| Interval between CT and MRE (mo) | 3.2 ± 0.6 (1 - 6) |
| HBP signal intensity (iso/ slightly high/ high) | 31/ 18/ 3 |
| Liver stiffness (kPa, Av ± SD, range) | 6.9 ± 3.2 (1.7 – 12.8, median 6.5) |
| ECV of GBFN (%, Av ± SD, range) | 31.2 ± 7.9 (14.6 – 50.4, median 30.7) |
| ECV of BGL (%, Av ± SD, range) | 36.2 ± 8.0 (22.6 – 53.2, median 35.8) |
| ECV ratio (Av ± SD, range) | 0.88 ± 0.19 (0.53 – 1.36, median 0.88) |

M/F: male/female, Av/SD: average/ standard deviation, mALBI: modified albumin-bilirubin, CP: Child -Pugh, GBFN: gallbladder fossa nodularity, MRE: MR elastography, HBP: hepatobiliary phase, ECV: extracellular volume fraction, BGL: background liver. ECV ratio is defined as ECV of GBFN divided by that of GBL.

Table 2 Details of the longitudinal change of the 10 patients for whom more than 3-year follow up was available.

| Liver function  GBFN grade | deteriorate | No change | improve |
| --- | --- | --- | --- |
| Up-grade | 0 | 1  G1→G3: 2b/ 5yrs¶ | 0 |
| No change | 2  G1: 2b→3/ 3yrs  G3: 2a→2b/ 6yrs | 1  *G2: 2b/ 3yrs | 0 |
| Down-grade | 2  G3→G1: 2a→3/ 8yrs† G2→G1: 2a→3/ 3yrs | 4  G2→G1: 2b/ 7yrs G2→G1: 2b/ 12yrs *G2→G0: 3/ 7trs G3→G1: 2b/ 8yrs‡ | 0 |

GBFN: gallbladder fossa nodularity, G: grade, yrs: years, *: female patient.

†: patient in Fig. 5

‡: patient in Fig. 6: This patient had a CT before 7a and another CT after 7c, therefore, total follow-period was 8 years.

¶: patient in Fig. 7

Information within the parentheses indicate those for each patient. The left-side, the center, and the right-side information within parentheses represent GBFN grade, liver function as expressed by modified ALBI grade, and duration of follow-up in years, respectively. For example, “G3→G1: 2a→3/ 8yrs” indicates GBFN grade changed from G3 to G1, and mALBI grade changed from 2a to 3, over 8-year follow-up.
